# Multi-Focus Image Fusion Method for Vision Sensor Systems via Dictionary Learning with Guided Filter

**DOI:** 10.3390/s18072143

**Published:** 2018-07-03

**Authors:** Qilei Li, Xiaomin Yang, Wei Wu, Kai Liu, Gwanggil Jeon

**Affiliations:** 1 College of Electronics and Information Engineering, Sichuan University, Chengdu 610064, China; qilei.li@outlook.com; 2 Fujian Provincial Key Laboratory of Information Processing and Intelligent Control, Minjiang University, Fuzhou 350121, China; 3 College of Electrical and Engineering Information, Sichuan University, Chengdu 610064, China; kailiu@scu.edu.cn; 4 School of Electronic Engineering, Xidian University, Xi’an 710071, China; ggjeon@gmail.com; 5 Department of Embedded Systems Engineering, Incheon National University, Incheon 22012, Korea

**Keywords:** vision sensor system, multi-focus image fusion, dictionary learning, guided filter

## Abstract

Vision sensor systems (VSS) are widely deployed in surveillance, traffic and industrial contexts. A large number of images can be obtained via VSS. Because of the limitations of vision sensors, it is difficult to obtain an all-focused image. This causes difficulties in analyzing and understanding the image. In this paper, a novel multi-focus image fusion method (SRGF) is proposed. The proposed method uses sparse coding to classify the focused regions and defocused regions to obtain the focus feature maps. Then, a guided filter (GF) is used to calculate the score maps. An initial decision map can be obtained by comparing the score maps. After that, consistency verification is performed, and the initial decision map is further refined by the guided filter to obtain the final decision map. By performing experiments, our method can obtain satisfying fusion results. This demonstrates that the proposed method is competitive with the existing state-of-the-art fusion methods.

## 1. Introduction

**Figure 1:**
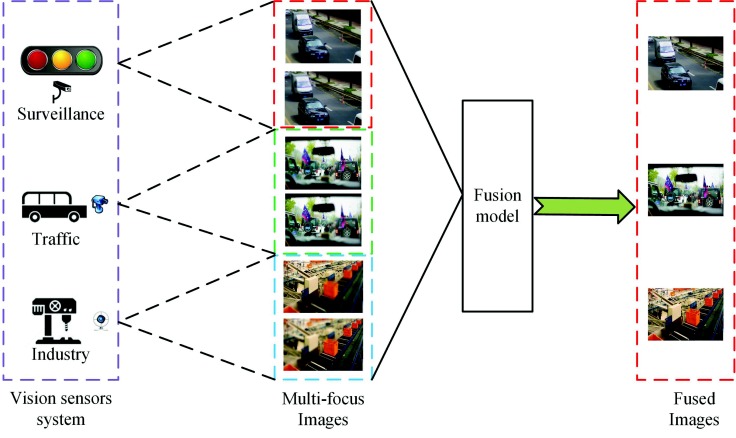
Applications of vision sensor systems (VSS) in real life.

A large number of images can be obtained via vision sensor systems (VSS). These images are employed in many applications, such as surveillance, traffic and industrial, as is shown in Figure 1. For example, these images can be used to build an urban surveillance system, as in [1]. Besides, these images can be utilized to monitor objects and behavior in [2]. Images with sufficient information are required to achieve these goals. However, Since the depth of field (DOF) is limited in vision sensors, it is hard to obtain an all-focused image, which can provide more information compared to the single multi-focus image. This causes difficulties for VSS in analyzing and understanding the image. In addition, it also causes redundancy in storage. To address those problems, multi-focus image fusion technology can fuse the complementary information from two or more defocused images into a single all-focused image. Compared with each defocused image, the fused image with extended DOF can provide more information and can thus better interpret the scene.

Of the popular multi-focus image fusion methods, there are two major branches [3]: spatial domain methods and transform domain methods.

Spatial domain methods directly fuse source images via specific fusion rules. The primitive way is to calculate the mean of the source images pixel by pixel. To avoid the same treatment of pixels, Tian et al. [4] used a normalized weighted aggregation approach. Li et al. [5] decomposed the source image into the detail layer and base layer, then fused them by using a guided filter. However, the pixel-based fusion methods are often subject to noise and misregistration. To further enhance the fusion performance, some block- and region-based methods have been proposed. For instance, Li et al. [6] chose the image blocks based on spatial frequency. Miao et al. [7] measured the activity of blocks based on image gradients. Song et al. [8] fused source images adaptively by using the weighted least squares filter. Jian et al. [9] decomposed images into multiple scales and fused them through a rolling guidance filter. Zuo et al. [10] fused images based on region segmentation. Besides spatial frequency and image gradients, the energy of Laplacian method is also an important method to evaluate the sharpness measures. Although the influences of noise and misregistration become smaller, those methods often suffer from block artifacts and contrast decrease.

Unlike the former, the main idea of transform domain methods is to fuse multi-focus images in the transform domain. Those methods include the Laplacian pyramid (LP) [11], the ratio of the low-pass pyramid (RP) [12], the gradient pyramid (GP) [13], discrete wavelet transform (DWT) [14], dual-tree complex wavelet transform (DTCWT) [15] and discrete cosine harmonic wavelet transform (DCHWT) [16]. Nowadays, some multi-scale geometry analysis tools are employed. For instance, Tessens et al. [17] used curvelet transform (CVT) to decompose multi-focus images. Zhang et al. [18] used nonsubsampled contourlet transform (NSCT) to decompose multi-focus images. Huang et al. [19] fused source images in the non-subsampled shearlet transform domain. Wu et al. [20] used the hidden Markov model to fuse multi-focus images. Besides the transform domain methods listed above, some new transform domain method such as independent component analysis (ICA) [21] and sparse representation (SR) [22,23] are also used to fuse multi-focus image. To avoid block effects and undesirable artifacts, those methods often employ the sliding window technique to obtain image patches. For instance, SR-based image fusion methods divide source images into patches via a sliding window with a fixed size and transform the image patches to sparse coefficients, then apply the L1-norm to the sparse coefficients to measure the activity level.

Although some of the multi-focus fusion methods perform well, there are still some drawbacks that remain to be settled. For spatial domain methods, some of them are subject to noisy and misregistration, and block effects may be caused in the fused images. Besides, some methods also result in increased artifacts near the boundary, decreased contrast and reduced sharpness. For transform domain methods, the fusion rules are based on the relevant coefficients; thus, a small change in the coefficients would cause a huge change in pixel values, which would cause undesirable artifacts.

Sparse representation [22] has drawn much attention in recent years for its outstanding ability in computer vision tasks and machine learning, such as image denoising [24], object tracking [25,26], face recognition [27] and image super-resolution [28,29,30]. Similarly, sparse representation has achieved great success in the field of multi-focus image fusion [31,32,33,34,35]. Yang et al. [31] brought SR to multi-focus image fusion. Based on this work, Liu et al. [32] fused the multi-focus images based on SR with adaptive sparse domain selection. In their method, different categories of images were utilized to learn multiple sub-dictionaries. However, this often leads to overfitting of the sub-dictionaries and causes obvious artificial effects. To address this problem, Liu et al. [33] decomposed source images into multiple scale and fused them by using SR. To further improve the resolution of the fused image, Yin et al. [34] combined image fusion and image super-resolution together based on SR. Besides, Mansour et al. [35] proposed a novel multi-focus image fusion method based on SR with a guided filter, and the Markov random field was also utilized to refine the decision map in their method. These methods can achieve good performances. However, there are still some drawbacks that remain to be settled:

1. Some SR-based methods [31,32,33,34,35] obtain the fused image by fusing the corresponding sparse coefficients directly, while a small change in the coefficients may cause a huge variation in pixel values. This would lead to undesirable effects on the fused image.
2. For some ambiguous areas in the multi-focus image, the sparse coefficients cannot determine if they are focused or not. This often causes spatial inconsistency problems. For example, the initial map obtained by Mansour’s method [35] suffered from spatial inconsistency. The following process to refine the decision map requires much computational cost.
3. The boundary between the focused area and the unfocused area is smooth, while the final decision map obtained by Mansour’s method [35] was sharp on the boundary. This may lead to halo effects on the boundary between the focused area and the unfocused area.

**Figure 2:**
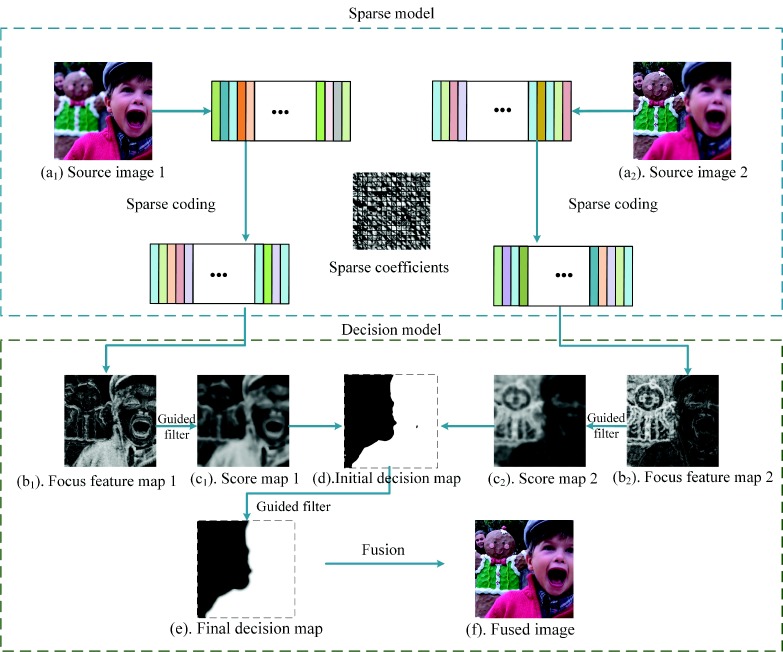
Framework of the proposed method.

To solve these problems, we propose a novel multi-focus image fusion method (SRGF) by using sparse coding and the guided filter [36]. The proposed method uses sparse coefficients to classify the focused regions and defocused regions to obtain the focus feature maps, as shown in Figure 2b. Then, the guided filter is used to calculate the score maps as shown in Figure 2c. An initial decision map as shown in Figure 2d can be obtained via comparing the score maps. After that, consistency verification is preformed, and the initial decision map is further refined by the guided filter to obtain the final decision map, as shown in Figure 2e. Compared with these traditional SR-based methods, there are three major contributions:

1. We use sparse coefficients to classify the focused regions and the unfocused regions to build an initial decision map, as shown in Figure 2d, rather than directly fusing the sparse coefficients. The initial decision map would be optimized in the latter steps. In this way, we avoid the artifacts caused by improper selection of the sparse coefficients.
2. To address the spatial inconsistency problem, we use the guided filter to smooth the focus feature maps, as shown in Figure 2b, fully considering the connection with the adjacent pixels. In this way, we effectively preserve the structure of images and avoid the spatial inconsistency problem.
3. To generate a decision map, which concerns the the boundary information, a guided filter is used to refine the initial decision map. By doing so, the boundary of the final decision map, as shown in Figure 2e, is smoothed, and it has a slow transition. Thus, the halo artifact of the fused image is efficiently reduced.

To validate the proposed method, we conduct a series of experiments. By the experiments, we demonstrate that the proposed method can obtain satisfying fusion results. Moreover, it is competitive with the existing state-of-the-art fusion method.

The remainder of paper is organized as follows. In Section 2, the SR theory and the guided filter are briefly reviewed. Section 3 describes the proposed multi-focus image fusion method in detail. Section 4 analyzes the experimental results. Finally, Section 5 concludes the paper.

## 2. Related Work

Basic theories of sparse coding and the guided filter are reviewed briefly in this section.

### 2.1. Sparse Coding

**Figure 3:**
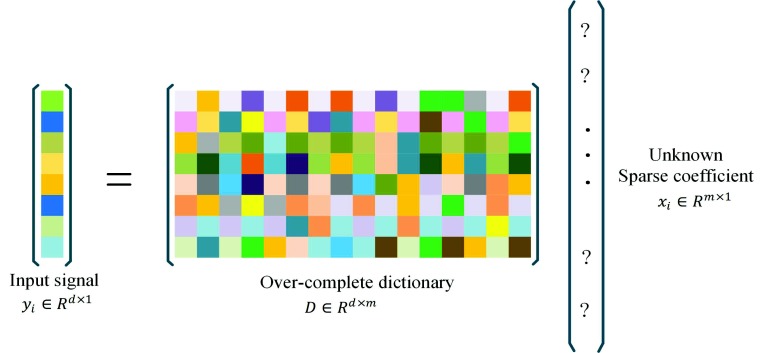
Sparse coding of a signal $y_{i}$.

Sparse signal coding [22] has drawn much attention in recent years for its outstanding ability in computer version tasks and signal processing. This is mainly because a signal can be composed into a dictionary and correlating sparse coefficients. In other words, given a set of *N* input signals $Y=\left\{y_{1},\cdot \cdot \cdot y_{N}\right\}\in R^{d\times N}$, each signal $y_{i}$ can be represented as:

(1) $$\min_{x_{i}}{∥y_{i}-Dx_{i}∥}_{2}^{2}\;s.t.{∥x_{i}∥}_{0}\leq k_{0},1\leq i\leq N$$

where $y_{i}\in R^{d},D\in R^{d\times m}$ is an over-complete dictionary, which has *M* atoms; $X={\left\{x_{i}\right\}}_{i=1}^{N},x_{i}\in R^{M}$ is the sparse coefficient of the input signal *Y*; *T* is a threshold of non-zero elements in each sparse coefficient. The basic concept is shown in Figure 3.

### 2.2. Guided Filter

GF [36] is an edge-preserving smoothing filter. It can avoid ringing artifacts since strong edges would not be blurred during the operation. In this paper, GF is used to smooth the focus feature maps and refine the decision map.

**Figure 4:**
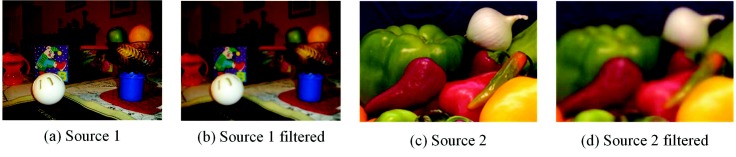
Two source images and the filtered images via the guided filter. The guidance images are the source images themselves, and parameters *r* and ϵ are set to three and 0.16, respectively.

Given an input image *P*, with a guidance image *I*, in a local window $\omega_{k}$, and pixel *k* being the central pixel, we assume that the resulting image *O* is linear correlated with *I*.

(2) $$O_{i}=a_{k}I_{i}+b_{k}\;\forall i\in \omega_{k}$$

where $\omega_{k}$ is a square window and its size is (2r+1)×(2r+1). To estimate the linear coefficients $a_{k}$ and $b_{k}$, the goal is to minimize the squared difference between *O* and *P*.

(3) $$E\left(a_{k},b_{k}\right)=\sum_{i\in \omega_{k}}\left({\left(a_{k}I_{i}+b_{k}-P_{i}\right)}^{2}+\epsilon a_{k}^{2}\right)$$

where ε is set manually. The following linear regression is used to calculate $a_{k}$ and $b_{k}$.

(4) $$\begin{array}{c} a_{k}=\frac{\frac{1}{\omega }\sum_{i\in \omega_{k}}I_{i}P_{i}-\mu_{k}{\bar{P}}_{k}}{\delta_{k}+\epsilon }\\ b_{k}={\bar{P}}_{K}-a_{k}\mu_{k} \end{array}$$

where |ω| means the count of pixels in a local window size of $\omega_{k}$. $\mu_{k}$ and $\sigma_{k}$ are the mean and variance of *I* in $\omega_{k}$ respectively. ${\bar{P}}_{k}$ is the mean of *P* in $\omega_{k}$. Output image *O* would be obtained according to Equation (2). The guided filter used for smoothing is shown in Figure 4.

## 3. Proposed Multi-Focus Image Fusion Method

In the proposed method, an over-complete dictionary is trained, and the correlating sparse coefficients are calculated. The coefficients would be used to measure the activity level, then the focus feature maps would be obtained according to the activity level. The guided filter is applied to the focus feature maps to generate the score maps. An initial decision map is obtained via comparing the score maps. Then, the guided filter is used for refining the initial decision map.

As shown in Figure 2, the proposed method can be divided into three parts:

1. Learning dictionary
2. Calculating the sparse coefficients and obtaining the initial decision map
3. Refining the initial decision map

The following subsections will introduce these steps mentioned above in detail.

### 3.1. Learning Dictionary

**Figure 5:**
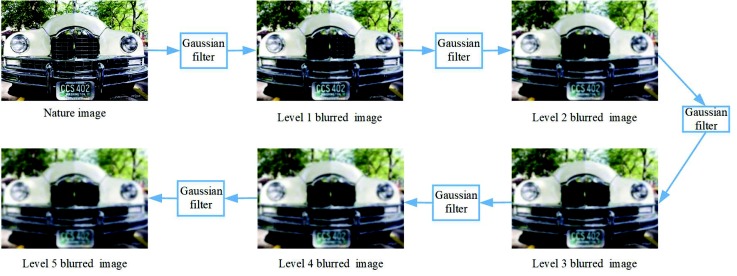
Nature images and filter results of a Gaussian filter with the standard deviation of three and a size of 5×5.

Considering the differences between the focused regions and defocused regions, we want to learn a dictionary that can perform well on both types. We blur the nature images several times using a Gaussian filter, since the blurred images have a similar visual effect as the defocused image patches; besides, we can control the blur level according to the actual needs. This process is shown in Figure 5.

**Figure 6:**
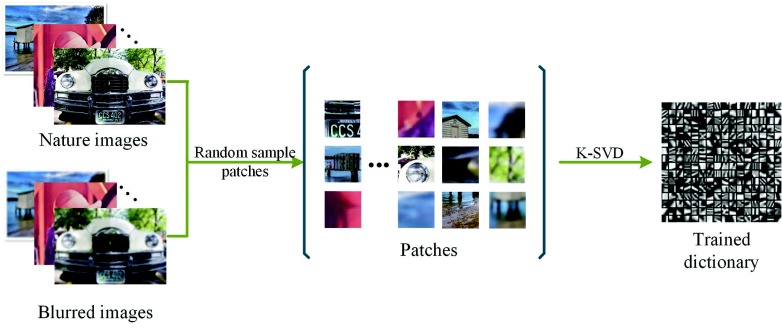
The general process of training a dictionary.

Next, many image patches of a fixed size would be randomly sampled from the nature images and the corresponding blurred images. This aims to extend the patch diversity [37] for a better sparse dictionary compared with traditional SR methods. Then, these will be used for learning the dictionary *D*, which can be calculated by solving Equation (1) via the K-SVD [22] algorithm. Figure 6 shows the general process.

To train the dictionary *D*, the related parameters are set as follows. The standard deviation and size of the Gaussian filter are set to three and 5×5, and the blur iteration number is set to five, respectively. The dictionary size is set to 64×512; the patch size is 8×8; the threshold of the non-zero numbers *T* is set to five. We randomly selected 10,000 patches from the source images to train the dictionary.

### 3.2. Sparse Coding and Obtaining Initial Decision Map

After the dictionary *D* is learned, it would be used for calculating the sparse coefficients of the *N* input multi-focus images. In the sparse coding phase, we adopt a sliding window with the same size as the patch size we adopted in the training phase (i.e., eight). Then, we use a sliding window to sample patches, from the source images pixel by pixel. When the patches are sampled, they will be expanded into column vectors $\hat{X_{i}}=\left\{x_{i1},x_{i2},\cdots ,x_{i(n-1)},x_{in}\right\}$, and the sparse coefficients will be calculated by solving Equation (5) via the OMP [38] algorithm.

(5) $${\hat{X}}_{i}=\min_{Xi}∥X_{i}{∥}_{1}\;s.t.{∥Y_{i}-DX_{i}∥}_{2}\leq \delta ,$$

where σ is a constant (it is set to 15 in this experiment) and $Y_{i},\left(0<i\leq n\right)$ is the input images. $\hat{X_{i}}=\left\{x_{i1},x_{i2},\cdots ,x_{i(n-1)},x_{in}\right\}$ (*n* denotes the number of patches). The output coefficients reflect if the input image patches are focused or not. An activity level measure function is set up as shown below:

(6) $$f\left(i\right)=∥{\hat{X}}_{i}{∥}_{0},\;0<i\leq n$$

**Figure 7:**
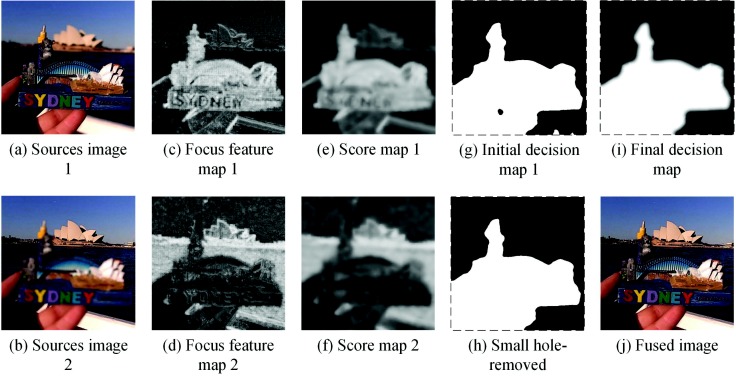
Obtaining the decision map and multi-focus image fusion.

Given the input multi-focus images $I_{1}$ in Figure 7a and $I_{2}$ in Figure 7b, the related activity level vector $f_{1}=\left(f_{11},f_{12},\cdots ,f_{1(n-1)},f_{1n}\right),f_{2}=\left(f_{21},f_{22},\cdots ,f_{2(n-1)},f_{2n}\right)$, can be calculated via Equation (6). The focus feature maps $E_{i},i\in \left\{1,2\right\}$ can be calculated by reshaping the related activity level measure vector $f_{i},i\in \left\{1,2\right\}$ as follows:

(7) $$E_{i}=reshape\left(f_{i}\right),\;i\in \left\{1,2\right\}$$

The focus feature maps are shown in Figure 7c,d. Since the difference between focused regions and defocused regions in $E_{i}$ is not obvious, GF is adopted to smooth the focus feature map. The score maps can be obtained as follows:

(8) $$S_{i}=GF\left(E_{i},E_{i},r_{1},\epsilon_{1}\right),i\in \left\{1,2\right\}.$$

where *GF* (•) represents the guided filter operator; the guidance images of the guided filter are focus feature maps themselves; and the parameters are set as $r_{1}=8,\epsilon_{1}=0.16$, respectively. The score maps are shown in Figure 7e,f.

After obtaining the score maps, the initial decision map can be calculated as follows:

(9) $$Q_{i}\left(x,y\right)=\left\{\begin{array}{cc} 1 & S_{1}\left(x,y\right)>S_{2}\left(x,y\right)\\ 0 & \mathrm{else} \end{array}\right.$$

### 3.3. Refining the Decision Map

The initial decision map $Q_{i}$ obtained by comparing the score maps may lead to some non-smoothing edges and some small holes, as shown in Figure 7g. This is because some regions have a similar visual effect on both input images, and the sparse coefficient cannot determine if they are focused or not. To remove those small holes, the small region remove strategy is adopted in our proposed method. The decision map after applying this strategy is shown in Figure 7h. Many small holes have been removed obviously. Then, the decision map would be up-sampled to the size as input images. This process can be expressed as follows:

(10) $$\begin{array}{c} Q_{r}\left(x,y\right)=Q_{i}\left(x,y\right)-small\;holes\\ Q\left(x,y\right)=upsample(Q_{r}\left(x,y\right)) \end{array}$$

In addition, the boundary between the focused area and the unfocused area is smooth, while the decision map *Q* is sharp on the boundary. To address this problem, the guided filter is adopted to optimize the decision map *Q*. In this section, we fuse the multi-focus images using decision map *Q*, then the fused image would be served as the guidance image of the guided filter. This process can be described according to the equation below:

(11) $$\begin{array}{c} W=Q\left(x,y\right)I_{1}\left(x,y\right)+\left(1-Q\left(x,y\right)\right)I_{2}\left(x,y\right)\\ \bar{Q}=GF\left(Q,W,r_{2},\epsilon_{2}\right) \end{array}$$

where *GF* (•) represents the the guided filter operator and the two parameters *r* and ϵ are set to eight and 0.1, respectively. The filtered result of the decision map is shown in Figure 7i.

### 3.4. Fusion

Finally, the fused image *F* can be obtained by:

(12) $$F\left(x,y\right)=\bar{Q}\left(x,y\right)I_{1}\left(x,y\right)+\left(1-\bar{Q}\left(x,y\right)I_{2}\left(x,y\right)\right)$$

Figure 7j shows the fused image of the given source images.

## 4. Experiments

**Figure 8:**
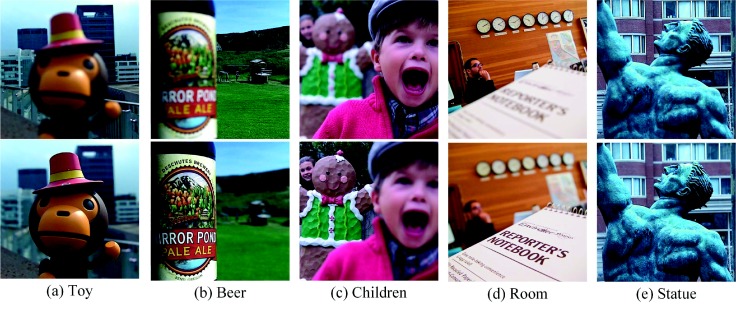
Portion of the test images in the “Lytro” dataset.

To verify the proposed method, we performed experiments on twenty groups of colorful multi-focus images selected from the image dataset “Lytro” [35]. The size of all test images is 520×520. Part of the test images is shown in Figure 8.

The proposed method is compared with some popular methods, such as DTCWT [15], CVT [17], GFF [5], NSCT [18], SR [31], NSCT-SR [33] and CSR [39]. The parameters of those methods are set according to related publications.

To evaluate the proposed method objectively, four representative evaluation metrics are adopted as follows:

- Mutual information MI [40] measures how much information from the source images the fused image contains. When the value of MI is high, it indicates that the fused image contains more information from the source images.
- Edge retention $Q^{AB/F}$ [41] calculates how much edge information transferred from the input images to the fused image. When the value of $Q^{AB/F}$ is high, it indicates that the fused image contains more edge information from the source images. The ideal value is 1.
- Feature mutual information FMI [42] is a non-reference objective image fusion metric that calculates the amount of feature information, like gradients and edges, existing in the fused image. When the value of FMI is high, it indicates that the fused image contains more feature information from the source images. The ideal value is 1.
- The standard deviation SD is used to measure the contrast in the fused image. When the value of SD is high, it indicates that the contrast of the fused image is higher.

To evaluate the fusion performance, the colorful images are transformed to gray images. For all these quality evaluation metrics, the larger value denotes the better performance. Moreover, the largest values are shown in bold.

### 4.1. Fusion of Multi-Focus “Face” Images

**Figure 9:**
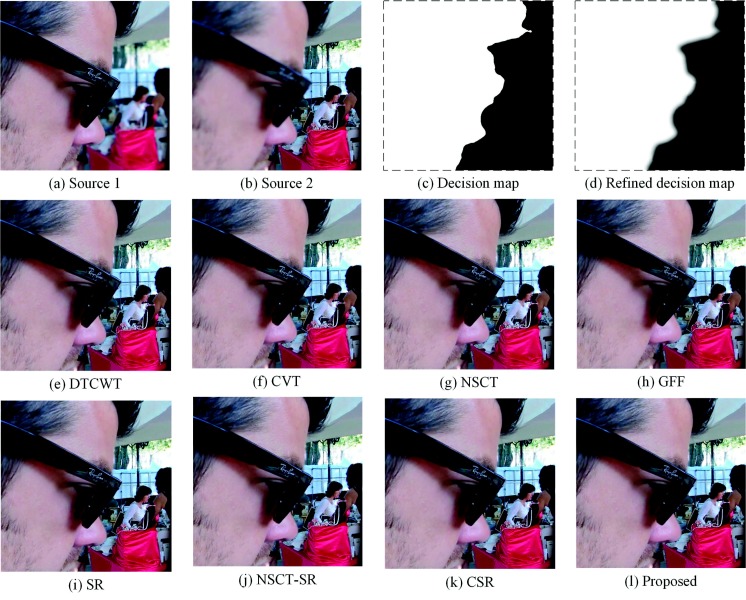
Fusion of “face” images. DTCWT, dual-tree complex wavelet transform; CVT, curvelet transform; NSCT, nonsubsampled contourlet transform; SR, sparse representation.

Experiments are performed on the “face” images. As Figure 9a,b shows, Source Image 1 is focused on the left part; on the contrary, Source Image 2 is focused on the right part. The man’s face and glasses separate the focused region and defocused region. The decision map and the refined decision map are shown in Figure 9c,d; the decision map separates the boundary of the focused region and the defocused region precisely. The fused result by the proposed method is shown in Figure 9l. Figure 9e–k shows the fused results of the DTCWT-, CVT-, NSCT-, GFF-, SR-, NSCT-SR- and CSR-based methods, respectively. As Figure 9 shows, the fused results make full use of the two source images. Compared with the DTCWT, CVT and NSCT methods, the proposed method produces an edge-smoothing fused image. Besides, the quantitative assessments are shown in Table 1. Bold denotes the largest value. The glasses in Figure 9f,k are not clear enough. This is mainly because of the CVT method and CSR method losing some edge information of the source images. This also leads to a low score in $Q^{AB/F}$. Besides, the fused results obtained by the DTCWT method and NSCT method suffer a slight color distortion. The MI and FMI scores for the two fusion results are relatively low. This is because much spatial information is lost during the image decomposition process. The other methods, namely the GFF-, SR- and NSCT-SR-based methods, work well in visual observation. Combining Figure 9 and Table 1, the superiority of the proposed method is demonstrated.

### 4.2. Fusion of Multi-Focus “Golf” Images

**Figure 10:**
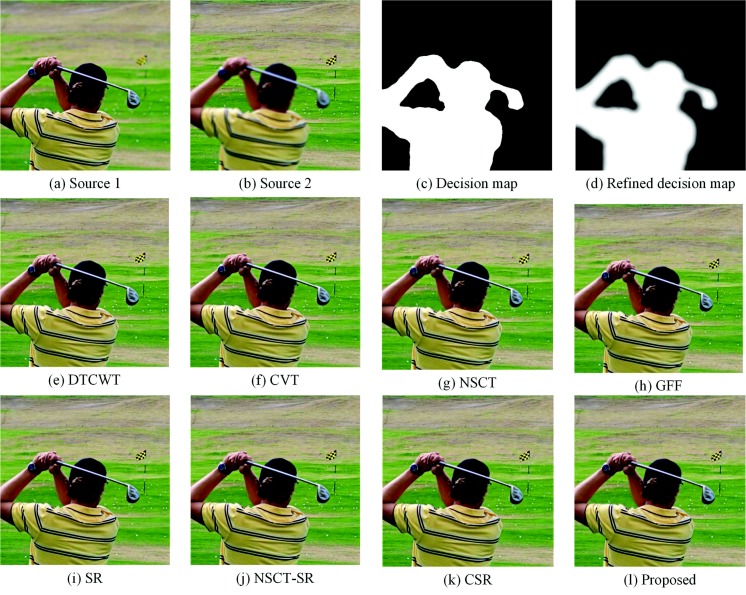
Fusion of “golf” images.

In this part, experiments are performed on “golf” images, as shown in Figure 10a,b. Source Image 1 is focused on the man and the golf club, while Source Image 2 is focused on the background. The two regions are separated by the decision map shown in Figure 10c,d. The fusion result obtained by the proposed method is shown in Figure 10l. Figure 10e–k shows the fused results of the DTCWT-, CVT-, NSCT-, GFF-, SR-, NSCT-SR- and CSR-based methods, respectively. The quantitative assessments are shown in Table 2. It can be seen that the ringing effect around the edge of the DTCWT-based and CVT based methods is obvious. Besides, the contrast of the fused image is reduced at the edge of the hat. These are because of the inappropriate image decomposition level, and the fused coefficients of DTCWT and CVT cannot represent the edge information. The $Q^{AB/F}$ and SD scores for their fused images are pretty low. Besides, The results of the SR-based method and CSR-based method contain some “artifacts”. Some artificial edges are introduced in the T-shirt and the background. The GFF and NSCT methods yield some artifacts in the man’s hair. The result of our method has the best visual effects. Namely, the proposed method outperforms all comparative methods in both visual effects and evaluation indicators.

### 4.3. Fusion of Multi-Focus “Puppy” Images

**Figure 11:**
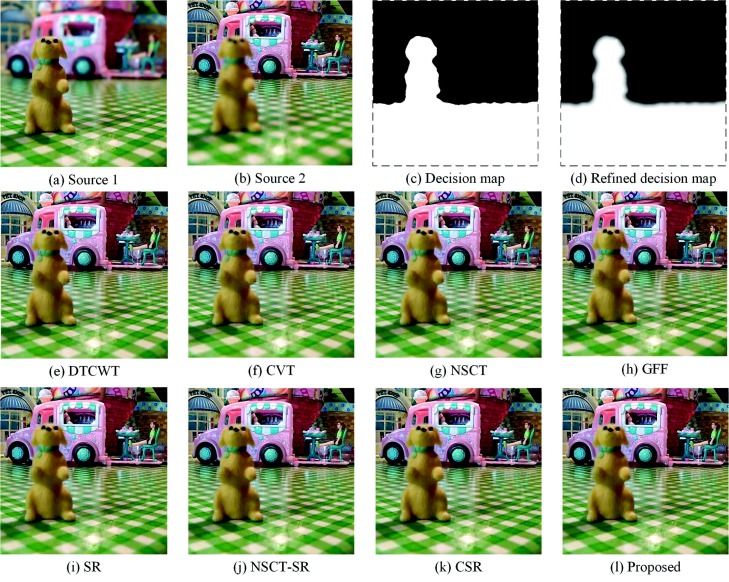
Fusion of “puppy” images.

Experiments are performed on the “puppy” images, as shown in Figure 11a,b. Source Image 1 is focused on the puppy and the foreground; Source Image 2 is focused on the background. The decision map and the refined decision map are shown in Figure 11c,d. The border between the focused region and the defocused region is obviously separated by the decision map. The proposed method fusion result is shown in Figure 11a. From Figure 11e–k, the fused results of the DTCWT-, CVT-, NSCT-, GFF-, SR-, NSCT-SR- and CSR-based methods, respectively. The quantitative assessment for this experiment is shown in Table 3. Compared with the proposed method, the DTCWT-, CVT- and NSCT-based methods choose irrational regions, which leads to unclear edges. For these methods, the quantitative assessments in terms of $Q^{AB/F}$ and FMI are relatively low. The fused images of the SR-based method and NSCT-SR-based method look better with respect to this issue, but there are still some small blocks in the fused images. This is mainly for the traditional SR-based methods using the sparse coefficients to fuse the multi-focus images, which often lead to block effects. The fused image of the GFF-based method performs well, but the contrast of the fused image is decreased due to the unsuitable proportion of the “detail layer” and “base layer”. The proposed method fusion result retains abundant information and handles the boundary well. Figure 11 and Table 3 demonstrate that the proposed method outperforms all comparative methods in this experiment.

### 4.4. Statistical Analysis of Fusion Results

**Figure 12:**
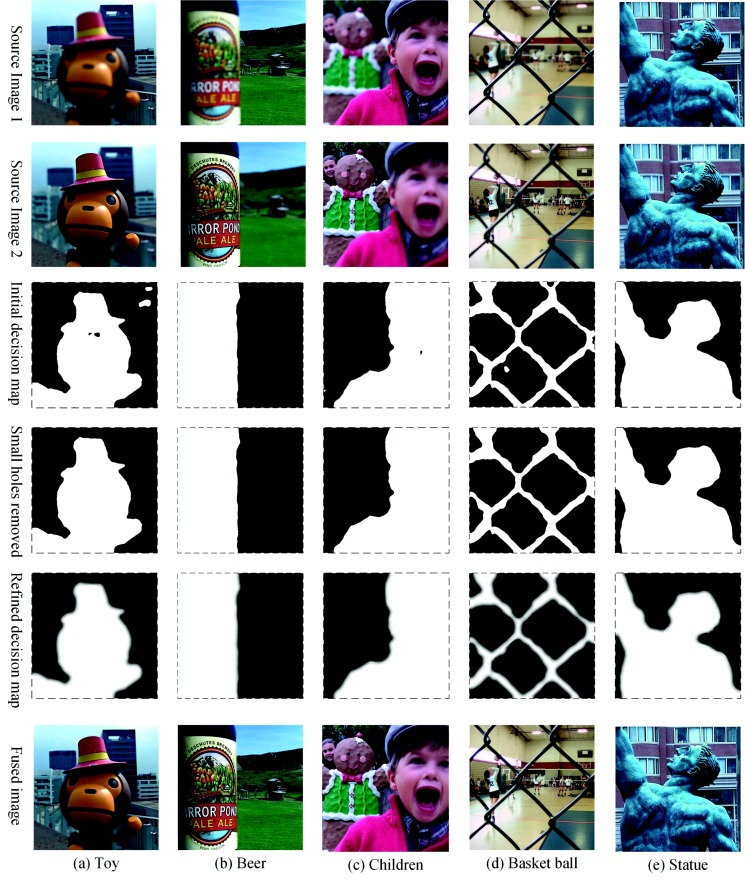
Fusion of a portion of the test images in the “Lytro” dataset.

Experiments were performed other images in the “Lytro” dataset. Some fusion results are shown in Figure 12. The proposed method can produce a precise decision map, which separates the focused region from the unfocused region accurately. Besides, the refined decision map obtained by the guided filter is robust to edges, which effectively avoids the artifacts on the edge.

To further demonstrate the effectiveness of our method, a one-way ANOVA test was performed to statistically compare the quantitative assessment distributions of all images in the “Lytro” dataset. The threshold of *p*-value was set to 0.05. Table 4 shows the results of the ANOVA test. Smaller values mean more significant differences. The *p*-values smaller than the threshold are shown in bold.

**Figure 13:**
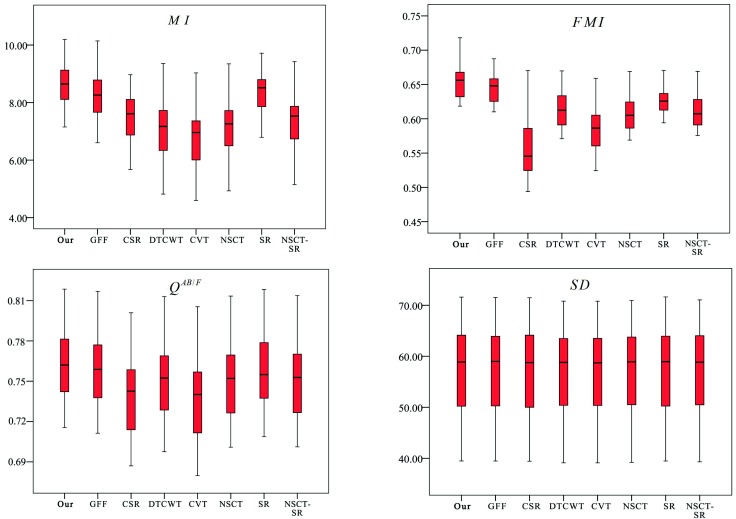
Boxplots of the statistical results.

It can be seen that the *p*-values for MI and FMI are smaller than the pre-defined threshold. This means that there are significant overall differences in MI and FMI. To figure out where these differences occurred, post hoc tests were performed on MI and FMI. The threshold of the *p*-value was also set to 0.05, and the post hoc test results are shown in Table 5. All values less than the threshold are bolded. It can be seen that there are significant differences between our methods and other methods in terms of MI and FMI. Moreover, the boxplots of the statistical results are shown in Figure 13. In terms of MI and FMI, the results obtained by our method have larger values and more concentrated distributions. In terms of $Q^{AB/F}$ and SD, our method has a slight advantage. The proposed method achieves slightly larger values, and the distribution is similar to other methods. According to the statistical results and the boxplots, it can be concluded that the proposed method can obtain significantly better results than other methods for MI and FMI and slightly better than other methods for $Q^{AB/F}$ and SD. In other words, the proposed method outperforms most of the existing fusion methods, and it achieves better performance.

### 4.5. Comparison of Computational Cost

To evaluate the required computation power of these methods, we evaluate the running time for each method. Table 6 shows the average running time for all the test images in the “Lytro” dataset. It can be seen that these SR-based methods (namely SR, NSCT-SR, CSR and SRGF) require more running time than other methods. That is due to the fact that calculating the sparse coefficients requires much computational cost. However, as we mentioned, it is obvious that the proposed method can achieve promising results. Besides, by using parallel computing with two threads and four threads, the running time is effectively reduced. This demonstrates that there is much room for improvement. On the one hand, we think it is tolerable to sacrifice a little time for a promising improvement. On the other hand, with the development of parallel computing and the wide use of the graphical processing unit (GPU), the time cost will be reduced soon. In our next work, we will further accelerate our method by using a GPU, which has many more cores than a CPU, to train the dictionary and to calculate the sparse coefficients.

## 5. Conclusions

In this paper, a novel multi-focus image fusion method is proposed. The proposed method utilizes sparse coefficients to produce focus feature maps, and the guided filter is used to generate an initial map and to refine the decision map. The decision map obtained by our method separates focused regions from defocused regions precisely. Compared to traditional SR-based methods, the proposed method avoids the block effect and produces an edge-preserving fusion result. By performing experiments, we demonstrate that the proposed method outperforms other popular approaches, and it is competitive with the state-of-the art image fusion method.

## Figures and Tables

**Table 1:** Quantitative assessments of “face” images.

Methods	DTCWT	CVT	NSCT	GFF	SR	NSCT-SR	CSR	SRGF
MI	7.9033	7.5579	7.9802	8.9431	8.8896	8.1653	8.2092	**9.3347**
$Q^{AB/F}$	0.7313	0.7141	0.7283	0.7419	0.7392	0.7294	0.7130	**0.7450**
FMI	0.6190	0.5790	0.6092	0.6517	0.6263	0.6125	0.5278	**0.6594**
SD	59.3473	59.3677	59.4553	59.5281	59.4959	59.2802	59.3981	**59.5499**

**Table 2:** Quantitative assessments of “golf” images.

Methods	DTCWT	CVT	NSCT	GFF	SR	NSCT-SR	CSR	SRGF
MI	6.5533	6.2184	6.7132	7.3211	7.0351	6.9582	6.6367	**7.5833**
$Q^{AB/F}$	0.7546	0.7396	0.7571	0.7613	0.7564	0.7583	0.7448	**0.7658**
FMI	0.6397	0.6122	0.6365	0.6597	0.6328	0.6405	0.5836	**0.6660**
SD	39.1174	39.1125	39.2127	39.4650	39.4650	39.3129	39.4160	**39.4795**

**Table 3:** Quantitative assessments of “puppy” images.

Methods	DTCWT	CVT	NSCT	GFF	SR	NSCT-SR	CSR	SRGF
MI	5.4492	5.3010	5.6032	6.8045	6.7931	5.9459	6.2977	**7.5010**
$Q^{AB/F}$	0.7617	0.7555	0.7625	0.7735	0.7713	0.7643	0.7618	**0.7771**
FMI	0.6229	0.6097	0.6220	0.6560	0.6387	0.6259	0.6013	**0.6767**
SD	46.6263	46.6002	46.7993	47.4477	47.2562	47.1882	47.3329	**47.5366**

**Table 4:** ANOVA test for the quantitative assessment distributions of the “Lytro” dataset.

Methods	Sum of Squares	F-Value	*p*-Value
MI	198.053	9.869	**0.000**
$Q^{AB/F}$	0.166	1.812	0.089
FMI	0.324	17.777	**0.000**
SD	11,717.513	0.001	1.000

**Table 5:** Post hoc tests for MI and FMI for the proposed method.

Metrics	*MI*	*FMI*
Methods	*p*-value	*p*-value
GFF	0.185	0.437
CSR	**0.000**	**0.000**
DTCWT	**0.000**	**0.001**
CVT	**0.000**	**0.000**
NSCT	**0.000**	**0.000**
SR	0.308	**0.027**
NSCT-SR	**0.000**	**0.000**

**Table 6:** Average running time for all the test images in the “Lytro” dataset (×1,×2,×4 denote the number of threads used for parallel computing).

Methods	DTCWT	CVT	NSCT	GFF	SR	NSCT-SR	CSR	SRGF (×1)	SRGF (×2)	SRGF (×4)
Running time (S)	1.012	1.841	6.4687	1.181	60.226	42.739	105.692	120.300	67.671	43.023

## References

[1] Pavlidis I., Morellas V., Tsiamyrtzis P., Harp S. (2001). Urban surveillance systems: From the laboratory to the commercial world. Proc. IEEE.

[2] Hu W., Tan T., Wang L., Maybank S. (2004). A survey on visual surveillance of object motion and behaviors. IEEE Trans. Syst. Man Cybern. Part C Appl. Rev..

[3] Stathaki T. (2008). Image Fusion: Algorithms and Applications.

[4] Tian J., Chen L., Ma L., Yu W. (2011). Multi-focus image fusion using a bilateral gradient-based sharpness criterion. Optics Commun..

[5] Li S., Kang X., Hu J. (2013). Image fusion with guided filtering. IEEE Trans. Image Process..

[6] Li S., Kwok J.T., Wang Y. (2001). Combination of images with diverse focuses using the spatial frequency. Inf. Fusion.

[7] Miao Q., Wang B. (2005). A novel adaptive multi-focus image fusion algorithm based on PCNN and sharpness. Sensors, and Command, Control, Communications, and Intelligence (C3I) Technologies for Homeland Security and Homeland Defense IV.

[8] Song Y., Wu W., Liu Z., Yang X., Liu K., Lu W. (2016). An adaptive pansharpening method by using weighted least squares filter. IEEE Geosci. Remote Sens. Lett..

[9] Jian L., Yang X., Zhou Z., Zhou K., Liu K. (2018). Multi-scale image fusion through rolling guidance filter. Futur. Gener. Comput. Syst..

[10] Zuo Y., Liu J., Bai G., Wang X., Sun M. (2017). Airborne infrared and visible image fusion combined with region segmentation. Sensors.

[11] Burt P.J., Adelson E.H. (1983). The Laplacian pyramid as a compact image code. Read. Comput. Vis..

[12] Toet A. (1989). Image fusion by a ratio of low-pass pyramid. Pattern Recognit. Lett..

[13] Petrovic V.S., Xydeas C.S. (2004). Gradient-Based Multiresolution Image Fusion.

[14] Li H., Manjunath B.S., Mitra S.K. Multisensor image fusion using the wavelet transform. Proceedings of the 1st International Conference on Image Processing.

[15] Lewis J.J., O’Callaghan R.J., Nikolov S.G., Bull D.R., Canagarajah N. (2007). Pixel- and region-based image fusion with complex wavelets. Inf. Fusion.

[16] Kumar B.K.S. (2013). Multifocus and multispectral image fusion based on pixel significance using discrete cosine harmonic wavelet transform. Signal Image Video Process..

[17] Tessens L., Ledda A., Pizurica A., Philips W. Extending the depth of field in microscopy through curvelet-based frequency-adaptive image fusion. Proceedings of the 2007 IEEE International Conference on Acoustics, Speech and Signal Processing.

[18] Zhang Q., Guo B.L. (2009). Multifocus image fusion using the nonsubsampled contourlet transform. Signal Process..

[19] Huang Y., Bi D., Wu D. (2018). Infrared and visible image fusion based on different constraints in the non-subsampled shearlet transform domain. Sensors.

[20] Wu W., Yang X., Pang Y., Peng J., Jeon G. (2013). A multifocus image fusion method by using hidden Markov model. Opt. Commun..

[21] Gao H. A simple multi-sensor data fusion algorithm based on principal component analysis. Proceedings of the 2009 ISECS International Colloquium on Computing, Communication, Control, and Management.

[22] Aharon M., Elad M., Bruckstein A. (2006). K-SVD: An Algorithm for Designing Overcomplete Dictionaries for Sparse Representation. IEEE Trans. Signal Process..

[23] Yang X., Wu W., Liu K., Chen W., Zhang P., Zhou Z. (2017). Multi-sensor image super-resolution with fuzzy cluster by using multi-scale and multi-view sparse coding for infrared image. Multimed. Tools Appl..

[24] Li H., Liu F. Image denoising via sparse and redundant representations over learned dictionaries in wavelet domain. Proceedings of the 2009 Fifth International Conference on Image and Graphics.

[25] Guha T., Ward R.K. (2012). Learning Sparse Representations for Human Action Recognition. IEEE Trans. Pattern Anal. Mach. Intell..

[26] Lu W., Bai C., Kpalma K., Ronsin J. Multi-object tracking using sparse representation. Proceedings of the IEEE International Conference on Acoustics, Speech and Signal Processing.

[27] Wright J., Yang A.Y., Ganesh A., Sastry S.S., Ma Y. (2009). Robust face recognition via sparse representation. IEEE Trans. Pattern Anal. Mach. Intell..

[28] Yang X., Wu W., Liu K., Chen W., Zhou Z. (2018). Multiple dictionary pairs learning and sparse representation-based infrared image super-resolution with improved fuzzy clustering. Soft Comput..

[29] Li H., Yang X., Jian L., Liu K., Yuan Y., Wu W. (2018). A sparse representation-based image resolution improvement method by processing multiple dictionary pairs with latent Dirichlet allocation model for street view images. Sustain. Cities Soc..

[30] Wei S., Zhou X., Wu W., Pu Q., Wang Q., Yang X. (2018). Medical image super-resolution by using multi-dictionary and random forest. Sustain. Cities Soc..

[31] Yang B., Li S. (2010). Multifocus image fusion and restoration with sparse representation. IEEE Trans. Instrum. Meas..

[32] Liu Y., Wang Z. Multi-focus image fusion based on sparse representation with adaptive sparse domain selection. Proceedings of the 2013 Seventh International Conference on Image and Graphics.

[33] Liu Y., Liu S., Wang Z. (2015). A general framework for image fusion based on multi-scale transform and sparse representation. Inf. Fusion.

[34] Yin H., Li S., Fang L. (2013). Simultaneous image fusion and super-resolution using sparse representation. Inf. Fusion.

[35] Nejati M., Samavi S., Shirani S. (2015). Multi-focus image fusion using dictionary-based sparse representation. Inf. Fusion.

[36] He K., Sun J., Tang X. (2010). Guided Image Filtering.

[37] Meng F., Yang X., Zhou C., Li Z. (2017). A sparse dictionary learning-based adaptive patch inpainting method for thick clouds removal from high-spatial resolution remote sensing imagery. Sensors.

[38] Pati Y.C., Rezaiifar R., Krishnaprasad P.S. Orthogonal matching pursuit: recursive function approximation with applications to wavelet decomposition. Proceedings of the 27th Asilomar Conference on Signals, Systems and Computers.

[39] Liu Y., Chen X., Ward R.K., Wang Z.J. (2016). Image fusion with convolutional sparse representation. IEEE Signal Process. Lett..

[40] Qu G., Zhang D., Yan P. (2002). Information measure for performance of image fusion. Electron. Lett..

[41] Xydeas C.S., Petrovic V. (2000). Objective image fusion performance measure. Electron. Lett..

[42] Haghighat M.B.A., Aghagolzadeh A., Seyedarabi H. (2011). A Non-Reference Image Fusion Metric Based on Mutual Information of Image Features.

